# A micro-flow, high-pH, reversed-phase peptide fractionation and collection system for targeted and in-depth proteomics of low-abundance proteins in limiting samples

**DOI:** 10.1016/j.mex.2023.102306

**Published:** 2023-07-31

**Authors:** Marta Zurawska, Mark Basik, Adriana Aguilar-Mahecha, Michal Dadlez, Dominik Domanski

**Affiliations:** a Mass Spectrometry Laboratory, Institute of Biochemistry and Biophysics, Polish Academy of Sciences, Warsaw, Poland; b Lady Davis Institute for Medical Research, Jewish General Hospital, Montreal, QC, Canada

**Keywords:** Proteomics, Two-dimensional peptide separation, Peptide fractionation, High-pH reversed phase liquid chromatography, Multiple reaction monitoring (MRM), Parallel reaction monitoring (PRM), Targeted proteomics, Global proteomics, Cell-cycle, CDK4/6 inhibitors, Micro-flow high-pH reversed-phase LC system for peptide fractionation and collection

## Abstract

We present a method and a simple system for high-pH RP-LC peptide fractionation of small sample amounts (30–60 µg), at micro-flow rates with micro-liter fraction collection using ammonium bicarbonate as an optimized buffer for system stability and robustness. The method is applicable to targeted mass spectrometry approaches and to in-depth proteomic studies where the amount of sample is limited. Using targeted proteomics with peptide standards, we present the method's analytical parameters, and potential in increasing the detection of low-abundance proteins that are difficult to quantify with direct targeted or global LC-MS analyses. This fractionation system increased peptide signals by up to 18-fold, while maintaining high quantitative precision, with high fractionation reproducibility across varied sample sets. In real applications, it increased the detection of targeted endogenous peptides by two-fold in a 25 cell-cycle-control protein panel, and in-depth MS analyses of nuclear extracts, it allowed the detection of up to 8,896 proteins with 138,417 peptides in 24-concatenated fractions compared to 3,344 proteins with 23,093 peptides without fractionation. In a relevant biological problem of CDK4/6-inhibitors and breast cancer, the method reproduced known information and revealed novel insights, highlighting that it can be successfully applied in studies involving low-abundance proteins and limited samples.

• Tested nine high-pH buffer/solvent systems to obtain a robust, effective, and reproducible micro-flow fractionation method which was devoid of commonly encountered LC clogging/pressure issues after months of use.
• Peptide enrichment method to improve detection and quantitation of low-abundance proteins in targeted and in-depth proteomic studies.
• Can be applied to diverse protein samples where the available amount is limited.

## Graphical abstract

**Figure:**
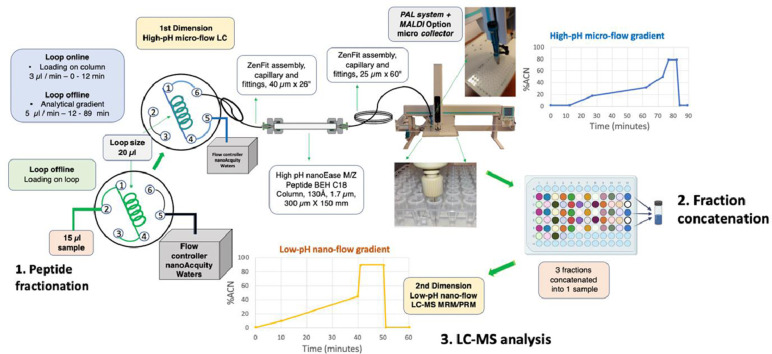

**Specifications Table**

**Table:** 

Subject area:	Biochemistry, Genetics and Molecular Biology
More specific subject area:	*Mass spectrometry-based proteomics*
Name of your method:	Micro-flow high-pH reversed-phase LC system for peptide fractionation and collection
Name and reference of original method:	Partly based on previous method: Kulak NA, Geyer PE, Mann M. Loss-less Nano-fractionator for High Sensitivity, High Coverage Proteomics. Mol Cell Proteomics. 2017 Apr;16(4):694–705. doi: 10.1074/mcp.O116.065136.
Resource availability:	https://skyline.ms/project/home/begin.view
	https://www.waters.com/nextgen/us/en/shop/columns/186009265-nanoease-m-z-peptide-beh-c18-column-300a-17-m-300-mm-x-150-mm-1.html
	https://www.ctc.ch/
	https://www.palsystem.com/

## Introduction

The study of low-abundance proteins by mass spectrometry (MS)-based proteomics entailing small sample amounts is a commonplace goal. For example, in cancer research, samples may provide only 10 s to 100 s of micrograms of total extracted proteins, and the aim is to measure cell signaling or cell-cycle protein components. To quantify low-abundance proteins accurately by MS, a strong analyte signal is required, and is often achieved through high-pH reversed-phase liquid chromatography (RP-LC) peptide fractionation, due to its high peak resolution and effective orthogonality of separation when combined with subsequent low-pH-RP LC-MS analysis [1]. Most high-pH fractionation methods start with large peptide input amounts (0.2 to 1 mg of digested proteins) and employ large column diameters (2.1–4.6 mm) and standard flowrates (0.1–1.0 mL/min) [2,3]. The selection of smaller bore columns and lower flowrates is an obvious choice to allow for the reduction of the required amount of sample, however, there is limited published evidence on the long-term use and robustness of high-pH RP peptide fractionation at nano- to micro-flow rates (0.3–10 µL/min.) and nano/capillary columns (75 to 300 µm i.d.) [4,5], and a lack of such commercially available systems. Furthermore, us and others have found that using the standard ammonium formate- (NH_4_FA) or ammonium hydroxide-based high-pH (pH: 9–10) buffers at micro- and nano-flow rates, often results in unstable system pressures and common precipitate formation and system clogging. We present a method and an easily obtainable system, based on a Waters Nano-LC and a PAL/CTC Analytics liquid-handling platform, for high-pH RP-LC peptide fractionation of small sample amounts (30–60 µg), at micro-flow rates with micro-liter fraction collection, using ammonium bicarbonate (NH_4_HCO_3_ - pH 8.5) as the optimized buffer for system stability and robustness. Ammonium bicarbonate buffer with its lower pH did not cause system clogging from the formation of precipitates and resulted in long-term system stability, allowing the fractionation of over 50 samples over a 4-month period using the same chromatographic column without system failure. The method is effective for both global and targeted proteomic analyses, demonstrating good retention time stability even between varied sample matrices, with a high quantitative precision. The method enabled the detection and quantitation of low-abundance proteins that were undetectable in non-fractionated samples even when using the most sensitive targeted analysis of parallel reaction monitoring (PRM) on the latest generation Orbitrap MS instrument (Exploris 480), revealing that this workflow has a useful potential in proteomic studies assessing low-abundance proteins in small sample amounts.

## Method Details

### Reagents and Chemicals

Mobile phases were prepared using LC-MS-grade acetonitrile (ACN) and water (J. T. Baker, Netherlands). Formic acid (FA), and ammonium bicarbonate, were purchased from Sigma-Aldrich (Saint Louis, USA)*.* MS-grade trypsin/Lys-C was purchased from Promega (# V5071, Fitchburg, WI).

### Equipment

The following equipment is required:

1st-dimension – High-pH RP-LC micro-flow fractionation

• Waters nanoAcquity UPLC (Waters, Milford, MA)
• HTS PAL instrument (LEAP Technologies/CTC Analytics AG, Switzerland)
• PAL MALDI Option (CTC Analytics AG, Switzerland)
• Waters nanoEase UPLC BEH130 C18 analytical column (300 µm X 150 mm *M/Z* Peptide, 1.7 µm particle size) (Waters, Milford, MA).
• 26-inch-long, 40 µm i.d. ZenFit capillary assembly, (Waters, Milford, MA)
• 60-inch-long, 25 µm i.d. ZenFit capillary assembly (Waters, Milford, MA)
• 96-well full-skirt PCR microplate (PCR-96-FS-C, Axygen, Corning Life Sciences, NY, US)

2nd-dimension low-pH nano-LC-MS – MRM/PRM/global analysis

• Waters nanoAcquity UPLC (Waters, Milford, MA)
• Waters Xevo triple-quadrupole (TQ) MS (Waters, Milford, MA)
• Evosep LC—One (Evosep Biosystems, Odense, Denmark)
• Orbitrap Exploris 480 MS (Thermo Fisher Scientific, Bremen, Germany)
• Waters nanoAcquity UPLC BEH130 C18 analytical column (75 µm × 150 mm, 1.7 µm particle size)
• Waters Symmetry 100 C18 trap-column (180 µm × 20 mm, 5 µm particle size) (Waters, Milford, MA)

### Software

High-pH fractionation collector

• PAL Cycle Composer Software CC 1.5.4, Agilent Chemstation Driver 2.1.3

### Proteomic Analysis

• Skyline-Daily Ver. 21 software (University of Washington, MacCoss Lab, Department of Genome Sciences, University of Washington)
• Proteome Discoverer version 2.4.0.305 (Thermo Fisher Scientific)

### Micro-flow High-pH Fractionation System Setup

#### PAL System

**Fig. 1:**
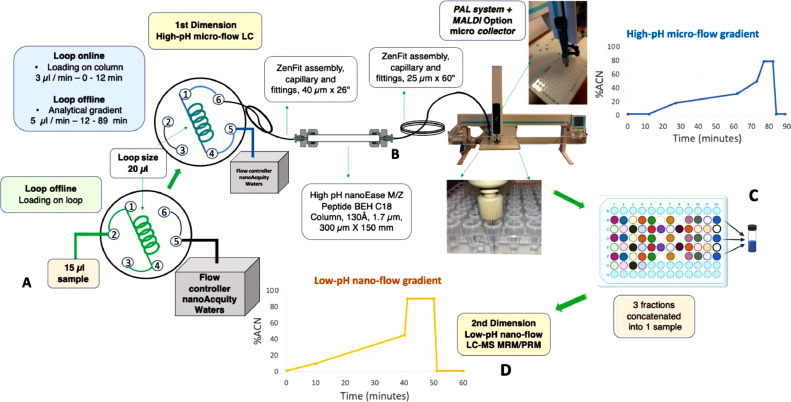
Schematic representation of the micro-flow high-pH fractionation system setup and nano-LC-MS-targeted analysis. (A) The protein digest is first separated using a micro-flow high-pH RP-LC system at a 5 µL/min flow rate using a Waters nanoAcquity UPLC, operated in a direct-injection mode. (B) Fractions (5 µL) are collected in a 96-well plate every 1 min using a CTC Analytics/LEAP Technologies PAL System with PAL MALDI Option configured as a fraction collector. (C) For increased analytical throughput, sixty-four fractions are concatenated into twenty-four pooled fractions. (D) One-third of each pooled fraction is analyzed by the second-dimension nano-flow low-pH RP-LC separation and either using MRM/PRM targeted analysis or global MS approaches. (For interpretation of the references to color in this figure legend, the reader is referred to the web version of this article.)

The high-pH micro-flow fractionation platform based on the PAL System can be easily implemented in any proteomics laboratory as all instrument components are commercially available. Peptides eluted from a high-pH micro-LC separation are fractionated into a 96-well plate using a PAL System with a PAL MALDI Option (LEAP Technologies/CTC Analytics AG, Switzerland) adjusted to collect microliter fractions into a PCR-microplate instead of spotting them on a MALDI plate (Fig. 1). This only requires minor modifications in the user software by entering correct plate and well positions (adjustment of the MALDI Tool Offset). The PAL System MALDI Tool was connected to the LC column with a 60-inch-long, 25 µm i.d. ZenFit capillary assembly (Waters, Milford, MA). The PAL fraction collector was operated via the Cycle Composer control software, setup as follows: PAL interface sign signal stand (TTL-In1), tray 01, tool offset *x* = 50.9 mm; tool offset *y* = - 900 mm; tool offset *z* = - 5,9 mm. A coated fused silica capillary, with a 360 mm OD, 20 µm ID x 39 mm, was used in the MALDI Tool dispensing holder as an elution needle for the deposition of fractions. This capillary was positioned to touch 30 µL of liquid (30% ACN) placed at the bottom of each well for 1 min to deposit a 5 µL fraction, and subsequently quickly retract while touching the side of the vessel to avoid removing any liquid before moving onto the next well. The needle is immersed, increasing the release of the fraction, and reducing cross-contamination between wells. Fractions were eluted into a 96-well full-skirt PCR microplate (PCR-96-FS-C, Axygen, Corning Life Sciences, NY, US).

### High-pH Reversed-phase LC Peptide Separation (1st-Dimension)

1) LC setup & Sample injection

For micro-flow high-pH RP peptide separation we employed a Waters nanoAcquity UPLC system with a Waters nanoEase *M/Z* Peptide 300 µm x 15 cm, high-pH stable C18 column using an end-capped, silica-based 1.7 µm particle, with a 130 Å pore size, with ‘Ethylene Bridged Hybrid (BEH) technology’, that had a usable pH range of 1–12. At a 5 µL/min flow rate, and a column temperature of 40 °C, normal pressure was around 6700 ± 300 psi at 2% ACN. The flow controller was connected to the rheodyne through an 18-inch-long, 25 µm i.d. capillary tubing (Waters, Milford, MA). The LC column was directly connected to the rheodyne through a 26-inch-long, 40 µm i.d. ZenFit capillary assembly, (Waters, Milford, MA).

The sample is loaded directly onto the chromatographic column without the use of any trap-column, reducing sample loss. With the rheodyne in the load position, the loop is offline, and the sample is loaded with the autosampler syringe. In our case, the loop was 20 µL and allowed to load 15 µL of sample. The sample is injected by switching the loop online and it is loaded onto the chromatographic column for 12 min at a 3 µL/min flow rate, with 2% ACN. Subsequently, the loop is switched offline, and the sample is separated by an ACN gradient at a 5 µL/min flow rate (Fig. 1A).

2) High-pH mobile phases

We tested nine combinations of mobile phase modifiers used in high-pH RP peptide fractionation, and all but one combination caused eventual clogging of the micro-flow LC system just with blank injections (Supplement 1). We determined that ammonium bicarbonate, with its lower pH of 8.5, used at 20 mM in phases A and B (with 20% water) resulted in stable system performance for a period of at least eight months. Although it is hard for us to prove this, one hypothesis is that the use of ammonium salt-based buffers at the higher-pressures encountered at nano/micro-flow rates, leads to decreased solubility and crystallization of the buffer, and subsequent column/system clogging, which is exacerbated at higher pH. Indeed, it is known that the solubility of ammonium salts in aqueous solution decreases as the pressure increases, and very high pressures (>14,000 psi) lead to nucleation and crystallization [6]. Potentially such high pressures can momentarily develop locally in the system.

The optimized phases were prepared using LC-MS grade solvents, with 20 mM ammonium bicarbonate in water as mobile phase A and 20 mM ammonium bicarbonate in 80% acetonitrile as mobile phase B. Mobile phases were prepared fresh before each fractionation, even if multiple fractionations were performed on the same day, to prevent ammonium bicarbonate from evaporating and causing a drop in pH. It must be ensured that ammonium bicarbonate is well dissolved.

3) Peptide fractionation and collection

For the 1st-dimension separation, 60 µg of digested proteins (practically 30–60 µg of digested peptides) was injected directly onto the analytical column as indicated above. The separation gradient was modified to optimize peptide distribution and consisted of a 5 µL/min flow rate, with the percentage of acetonitrile rising from 2% to 50% over 61 min, followed by column washing and re-equilibration. The entire 89 min LC method consisted of: 2.5% mobile phase B for 12 min, at a flow rate of 3 µL/min to allow the loading of the sample onto the column, followed by an increase in the flow rate to 5 µL/min and a linear gradient to 22.5% mobile phase B at 27 min, to 41% phase B at 62 min, and to 62.5% mobile phase B at 73 min, and then followed with 99% mobile phase B in 4 min and kept at 99% phase B for 5 min to wash, and re-equilibrated with 2.5% mobile phase B to 89 min. Eluted peptides were collected into 96-well plates, sealed with aluminum foil (VWR), frozen at −70 °C, dried using a SpeedVac concentrator, resuspended in 25 µL of 0.1% FA, 2% acetonitrile, sonicated for 5 min and centrifuged at 14,000 × g for 3 min. Samples were stored at −70 °C for further concatenation and low-pH nanoLC-MS analysis. The collected fractions were injected from the same microplate for the second dimension low-pH separation to minimize sample loss.

4) Concatenation

After the first-dimension separation, the 64 collected fractions in the 96-well plate were concatenated into 24 pooled fractions by combining the fractions as follows : 1, 25, 49; 2, 26, 50; 3, 27, 51; 4, 28, 52; 5, 29, 53; 6, 30, 54; and so forth [7]. Pooled fractions were frozen at −70 °C, dried using a SpeedVac concentrator, resuspended in 25 µL of 0.1% FA, 2% acetonitrile, sonicated for 5 min and centrifuged at 14,000 × g for 3 min. Samples were stored at −70 °C prior to low-pH nano-LC-MS analysis. From each fraction, one-third of the sample was injected for LC-MS analysis. The concatenation of fractions can be performed “on-the-fly” automatically by the PAL system with appropriate programing of its movements, or manually using a pipette for more flexibility.

### 2nd-dimension Low-pH RP Nano-LC-MS Analysis

For details of peptide separation and analysis in the second dimensions please refer to the Supplement 2. These are common LC-MS analytical procedures. In brief, a third of each fraction or of a pooled/concatenated fraction was injected by a Waters nanoAcquity UPLC or an Evosep system. The peptide separation component of the gradient on the analytical column in the second dimension lasted for approximately 40 min (nanoAcquity) or for 44 min on the Evosep. The nanoAcquity low-pH separation consisted of a nano-flow rate (350 nL/min) and water/acetonitrile phases with formic acid (0.1% v/v) as the modifier. The Evosep separation consisted of a nano-flow rate (500 nL/min) and water/acetonitrile phases with formic acid (0.1% v/v) as the modifier. The MS analyses consisted of either a targeted Multiple Reaction Monitoring (MRM)-MS analysis using a Waters Xevo TQ-MS, or a targeted Parallel Reaction Monitoring (PRM/SureQuant) analysis on an Exploris 480 Orbitrap system, in both cases using multiple internal peptide standards (Stable-isotope-labeled Internal peptide Standards; SIS) for each target protein being analyzed. Or an in-depth proteomic analysis, where the 10 most intense parent ions are fragmented and analyzed, performed on the Evosep-Exploris 480 Orbitrap system.

### Sample Preparation

The samples used in the method validation were prepared using common proteomic methods (see Supplement 2). Briefly, we used two types of samples, cell lines and patients derived xenografts (PDX).

Cell lysis and extraction of cytoplasmic and nuclear protein fractions from the cell lines, was performed using an NE-PER Nuclear and Cytoplasmic Extraction Kit (Thermo Scientific). Nuclear proteins were quantified using a BCA assay (Thermo Scientific).

The PDXs were lysed using a lysis buffer (see Supplement 2) and the extracted proteins were quantified using a BCA assay (Thermo Scientific).

One hundred micrograms of proteins from each sample were digested using the solid-phase-enhanced sample preparation for proteomics experiments (SP3) protocol [8].

After digestion, equal amounts of SIS were added to each sample and the peptide mixture was frozen and stored at −70 until used.

## Method Validation

1) Effectiveness of the micro-flow high-pH RP peptide fractionation system in increasing peptide signal intensity

**Fig. 2:**
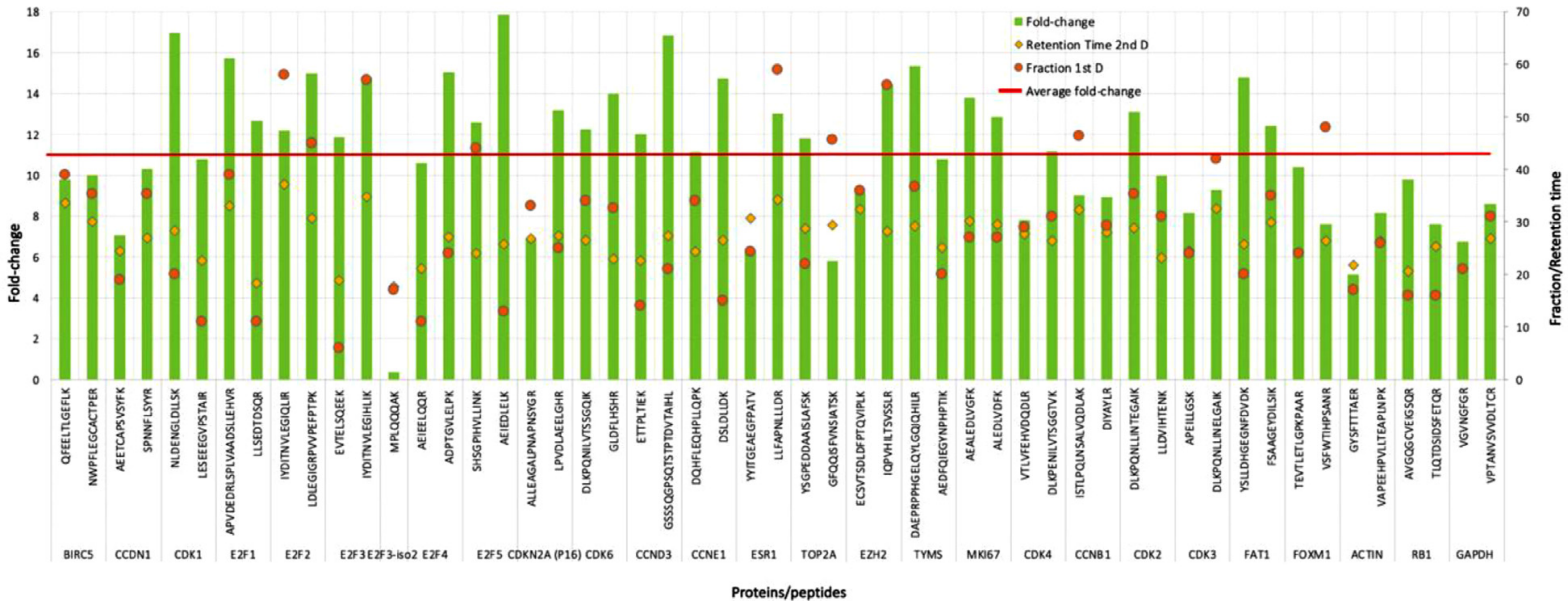
Signal enhancement for two peptides for each targeted protein, displayed as the fold-change of signal between the 2D LC-MRM-MS analysis and direct 1D LC-MRM-MS analysis. Green bars indicate the signal fold-change for each peptide. The fraction in which peptides elute in the high-pH 1st-dimension LC separation is indicated by yellow dots, while the retention time of the peptides in the low-pH 2nd-dimension LC separation are indicated by orange dots. The red line indicates the average signal fold-change. (For interpretation of the references to color in this figure legend, the reader is referred to the web version of this article.)

We evaluated the ability of the fractionation system to increase peptide signal intensity which is critical for the detection and quantitation of low-abundance proteins in MS analysis. For this, we used a multiple-reaction monitoring mass spectrometry (MRM-MS) quantitative assay targeting a panel of cell-cycle control proteins developed by us (See Supplementary Table 3). The SIS peptides employed in the assay provided the added benefit of being able to precisely assess many analytical criteria of the fractionation system. We evaluated the increase in signal intensity for the target peptides in a sample analyzed using the 2D-analysis (high-pH fractionation & low-pH LC-MRM-MS) compared to the same unfractionated sample analyzed directly by the 1-dimensional (1D) low-pH LC-MRM-MS analysis. This was done using a digested nuclear protein extract from an MCF-7 cell line with 142 SIS peptides (see supplement 3 for more details). Sixty µg of peptides were used per analysis on the 2D LC-MRM-MS system, and two µg (maximal load) of unfractionated digest was analyzed on the 1D LC-MRM-MS system (direct analysis). For each SIS peptide, we calculated the fold-change signal increase as the ratio between the SIS peptide peak area from the 2D LC-MRM-MS analysis and the SIS peptide peak area from the 1D LC-MRM-MS analysis (Fig. 2). The average obtained fold-change signal increase for the two best peptides per targeted protein was 11.0. A larger separation in the elution times of peptides between the two dimensions is an indication of higher orthogonality between the two LC separation methods, which can translate into an increase in signal and detectability of peptides.

2) Fraction concatenation strategy after peptide fractionation

**Fig. 3:**
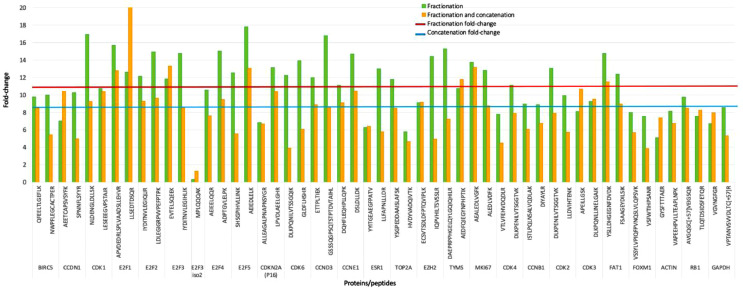
The effect of fraction concatenation on the peptide signal increase obtained from high-pH fractionation for the two best peptides for each protein. Twenty-four concatenated fractions are compared to 64 individual fractions in a targeted assay. Peptide signal increase is shown as a fold-change, calculated as the amount of SIS peptides after fractionation divided by the amount of SIS peptides in the corresponding non-fractionated sample. The signal increase was compared between samples just fractionated (average fold-change: 11.0) and the same sample after concatenation (average fold-change: 8.3). (For interpretation of the references to color in this figure legend, the reader is referred to the web version of this article.)

Concatenation is based on combining the early, middle, and late fractions eluted over equal time intervals, with little peptide overlap in the second dimension, into a single concatenated fraction [9], thus reducing the number of fractions requiring analysis in the second dimension and consequently reducing analytical time. To evaluate the efficiency of concatenation in maintaining the increase in peptide signal intensity obtained through fractionation, we evaluated three fractionated and concatenated replicates from an MCF-7 cell line nuclear extract. The fold-change signal increase was compared to the fold-change signal increase of the same peptides from the non-concatenated 2D LC-MRM-MS analysis. For the concatenated fractionation, the average fold-change signal increase compared to an unfractionated sample was 8.3 (Fig. 3). A slight loss in signal increase is sacrificed for the benefit of analysis time required in the second dimension.

3) Retention time stability

**Fig. 4:**
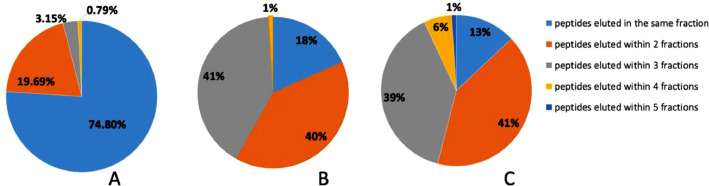
Retention time stability of the high-pH micro-flow fractionation system. The retention time stability of the target peptides was evaluated in three biological replicates of the same cell line (A), within three different cell lines (B), and within three different PDXs (C). (For interpretation of the references to color in this figure legend, the reader is referred to the web version of this article.)

The efficiency of a 2D LC separation system depends on a high peptide retention time stability (i.e., peptide/fraction elution reproducibility) in the high-pH dimension. We investigated the reproducibility of peptides eluting in the same fraction in the high-pH dimension as this would be beneficial for targeted proteomic analyses where only certain fractions containing the peptide targets are deemed useful for subsequent low-pH LC-MS analysis, to reduce analysis time and improve sample throughput. This stability was first examined using three biological replicates obtained from an MCF7 cell line analyzed a month apart. This analysis demonstrated that 74.8% of the peptides were eluted in the same fraction within the three replicates. The remaining 25.2% of peptides eluted in different fractions, from 1 up to 3 fractions from the main fraction (Fig. 4A). Considering that this type of system could be used to analyze and compare different cell lines or tissues to each other, it was important to evaluate the peptide retention stability when using slightly different matrices [10]. The same analysis was performed using three different cell lines (MCF10A, HCC-1143, and MDA-MB-468) and three patient-derived xenografts (PDXs). In the comparison of three cell lines, 18% of the peptides were eluted in the same fraction, 40% were eluted within two fractions, 41% were eluted within up to three fractions, and 1% were eluted in up to four fractions (Fig. 4B). Similar results were obtained by analyzing two PDXs of colorectal cancer and one ovarian cancer PDX; 13% of the peptides were eluted in the same fraction, 41% were eluted within two fractions, 39% were eluted within up to three fractions, 6% of the peptides were eluted within up to four fractions, and 1% were eluted in up to five fractions (Fig. 4C).

4) Quantitative reproducibility of the 2D-LC-MS analysis in targeted proteomics

We assessed the quantitative reproducibility of the entire workflow which includes the high-pH micro-flow fractionation with concatenation and nano-LC-PRM-MS targeted analysis, in measuring endogenous peptides from three technical inter-day replicates.

**Fig. 5:**
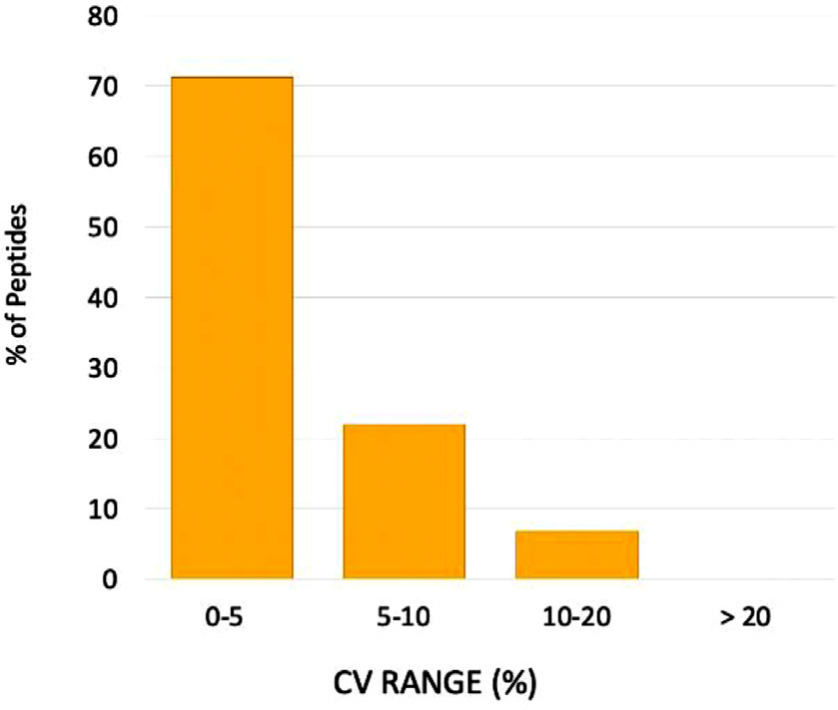
Quantitative reproducibility of the finalized workflow in a targeted proteomics application shown as a%CV distribution for measured endogenous peptides from three inter-day technical replicates. (For interpretation of the references to color in this figure legend, the reader is referred to the web version of this article.)

This was determinate using the PRM targeted approach with SIS peptides which increase the accuracy of quantitation. The precision in quantitation was defined by calculating the% coefficient of variation (%CV) for 73 individual quantified endogenous peptides in a pooled PDX sample. The measurement of the endogenous peptides was normalized to the SIS peptides added in equivalent amounts to each replicate. The majority of targeted peptides showed CVs of less than 5% (Fig. 5), 22% of the peptides presented a% CV between 5 and 10, and only 7% of peptides presented a%CV between 10 and 20. This indicates a very high and analytically acceptable precision of <20% in targeted proteomic studies, for all endogenous peptides detected, despite the noteworthy complexity of the analytical workflow.

5) Application of the 2D-LC-MRM/PRM-MS targeted analysis workflow to a study of cell-cycle proteins

To demonstrate the method's potential for broad applicability in the study of cell-cycle components or other low-abundance proteins, we used the workflow to analyze 25 protein targets potentially connected to CDK4/6-inhibitor resistance in cancer [20]. We analyzed two types of samples, an MCF-7 cell line and a colon cancer PDX, with and without fractionation. Out of 25 targeted proteins and 111 peptides, in the non-fractionated MCF-7 cell line sample, 16 proteins with 42 peptides were detected, compared to 22 proteins with 79 peptides after fractionation. Similarly, in the PDX analysis, 13 proteins with 40 peptides were detected without fractionation, compared to 23 proteins with 90 peptides after fractionation, out of 25 targeted proteins and 122 peptides (Table 1). This increase in detectability is significant and clearly a useful improvement even when using an already sensitive targeted analysis of PRM on the latest generation Orbitrap MS instrument (Exploris 480).

**Table 1:** Detected peptides and proteins in a targeted analysis of a 25 cell-cycle protein panel. Number of detected proteins and peptides in an MCF-7 cell line and a colorectal PDX with and without fractionation. Numbers in cells indicate the number of detected peptides per protein. The Protein Abundance Index (PAI) is defined as the ratio of the number of observed peptides to the number of observable peptides. PAI equal to 1 indicates that all the observable peptides are observed.

**Fig. 6:**
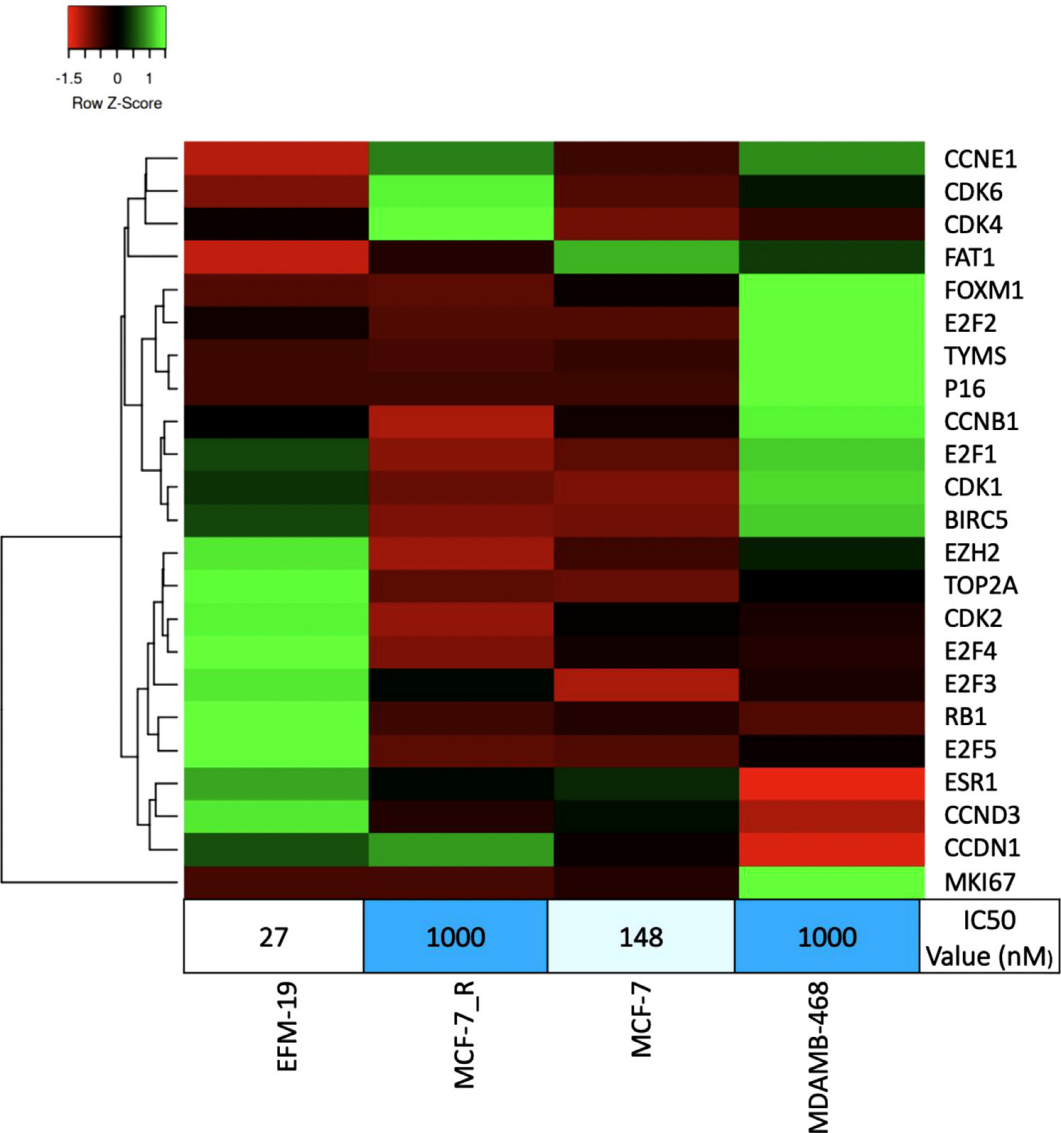
Levels of 23 targeted cell-cycle control proteins measured in four breast cancer cell lines using the 2D-LC-PRM-MS workflow. The heatmap was generated from the geometric means of multiple relative peptide levels (normalized to peptide standards) that identified each protein. The obtained values were normalized to the highest value of a given protein among the four cell lines analyzed. Heatmap parameters include: clustering method: average linkage; distance measurement method: euclidean [17]. (For interpretation of the references to color in this figure legend, the reader is referred to the web version of this article.)

We applied the developed workflow to study the levels of these proteins which could present resistance-related variations to CDK4/6-inhibitors [11]. Using this 25-protein panel (see Supplement 3), which was based on studies that showed a correlation between resistance to the treatment, we analyzed four breast cancer cell lines: EFM-19, MCF-7, MCF-7_Resistant (MCF-7_R), and MDA-MB-468. The MCF-7_R cell line was resistant to a CDK4/6-inhibitor (high IC50 value). Looking at the sensitive EMF-19 cell line and the resistant MDAMB-468 cell line, we see a completely different protein pattern. Furthermore, when we compare the resistant MCF-7_R cell line to the original sensitive MCF-7 cell line, we observe that the levels of CDK4, CDK6, cyclin D1 (CCDN1), cyclin E1 (CCNE1) and E2F-3 are significantly higher with a notable decrease in E2F4, RB1, FOXM1 and FAT1 (Fig. 6), consistent with previously published observations [12], [13], [14], [15], [16]. Our workflow is therefore able to confirm previous biological knowledge when measuring low-abundance proteins.

6) Increased proteome coverage in global/in-depth proteomic analyses

**Fig. 7:**
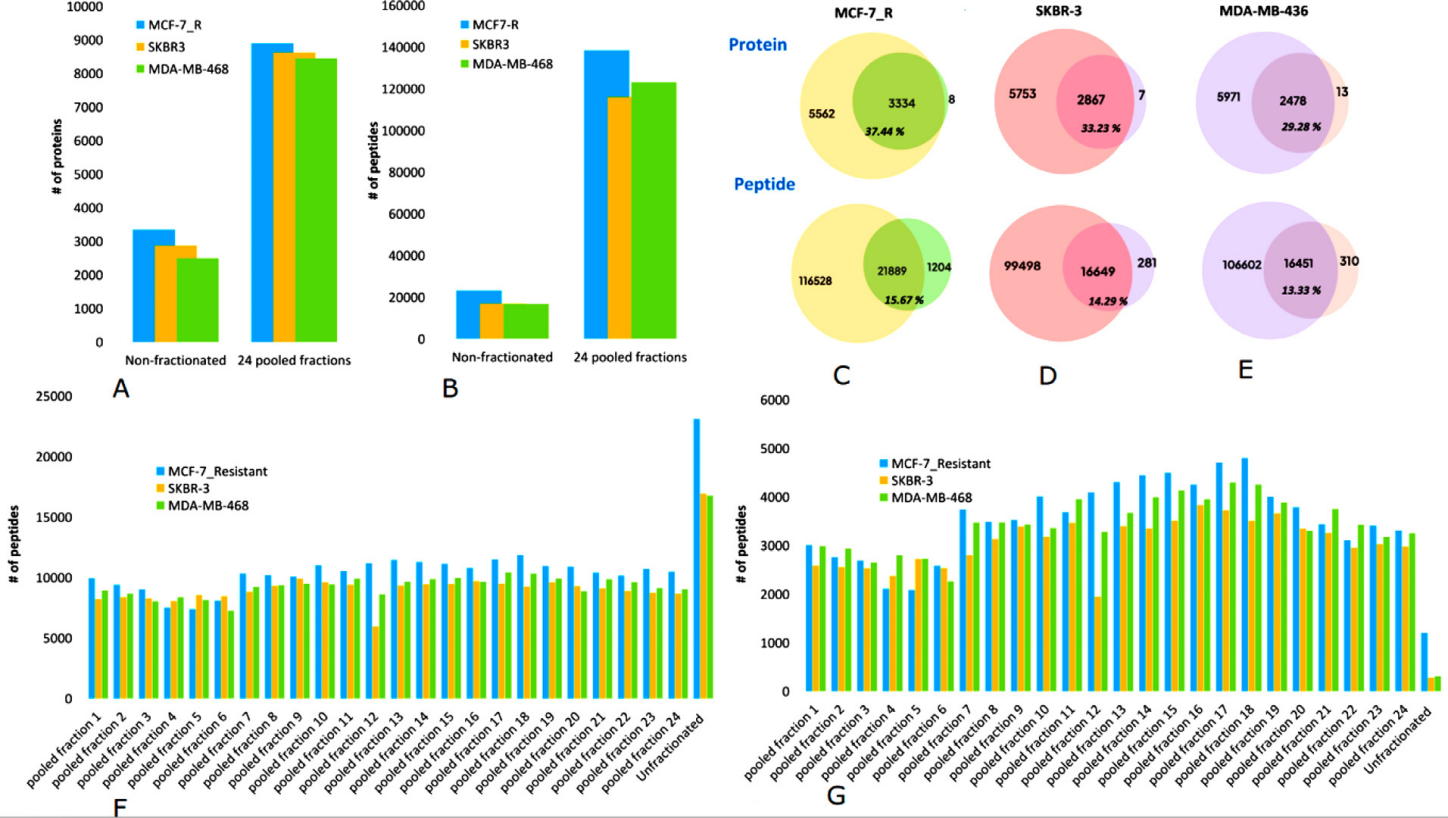
In-depth proteome analysis of three cell lines using the high-pH micro-flow fractionation method. Peptides from the MCF-7_R, SKBR-3, and MDA-MB-468 cell line nuclear extracts were used, comparing 24 concatenated fractions to the non-fractionated sample. Comparison of protein group identifications (A). Comparison of peptide identifications (B). Venn diagram analysis showing the overlap in the number of identified protein groups and peptides between the fractionated sample (left circles), non-fractionated sample (right circles), and overlap between the two samples (central circles) in the three cell lines (E). Distribution of peptides identified in each pooled fraction and in the respective non-fractionated sample (F). Distribution of unique peptides identified in each pooled fraction and in the respective non-fractionated sample (G). (For interpretation of the references to color in this figure legend, the reader is referred to the web version of this article.)

To further demonstrate the potential that our fractionation method presents, we performed an in-depth analysis of nuclear protein extracts from three different cell lines. Each cell line was fractionated and concatenated into 24 pooled samples. The total number of identified peptides in these pooled fractions was 138,417, 116,147 and 123,053 versus 23,093, 16,930 and 16,761 in the non-fractionated samples of the MCF-7_R, SKBR3 and MDA-MB-468 cell lines, respectively (Fig. 7B). These peptides correspond to 8896, 9620 and 8449 identified protein groups in the pooled fractions versus 3344, 2867 and 2478 in the non-fractionated samples for the MCF-7_R, SKBR3 and MDA-MB-468 cell lines, respectively (Fig. 7A). This indicates a 2.7-, 3.4-, and 3.4-fold increase, respectively, in the number of detected proteins, which is above a recently reported increase of 1.4-fold using a nano-flow fractionation system [4]. The overlap in the detected protein groups between the 24 pooled fractions and the non-fractionated sample was 3334 (37.4%), 2867 (33.2%) and 2478 (29.28%) proteins for the MCF-7_R, SKBR3 and MDA-MB-468 cell lines, respectively (Fig. 7C, D, E - protein). The overlap in the detected peptides between the 24 pooled fractions and the non-fractionated sample was 21,889 (15.8%), 16,649 (14.3%) and 16,451 (13.33%) peptides for the MCF-7_R, SKBR3 and MDA-MB-468 cell lines, respectively (Fig. 7C, D, E - peptide). A very small number of unique identifications in the non-fractionated samples indicates that the additional fractionation step loses a minimal number of identified proteins and peptides, some of which could also be within the allowed FDR error rate.

The concatenated fractions provided a nearly uniform distribution in the number of peptides across the set (Fig. 7F), and the number of unique peptide identifications (Fig. 7G) shows that the method allows to steadily identify unique peptides in each pooled fraction that are undetectable in the non-fractionated sample.

## Conclusions

A robust and effective, micro-flow, high-pH-RP-LC peptide fractionation and collection method was established, increasing the detection and quantitation of low-abundance proteins. This system can be easily implemented in proteomics laboratories from commercially available components, to allow for sensitive and in-depth analysis with small (30–60 µg) sample requirements.

## Ethics Statements

All procedures with patients’ specimens were performed under the principles of the 1964 Declaration of Helsinki and ethical standards of the local bioethical committee who permitted the PDX models development (decision 55/2017). All experimental procedures performed on mice during the PDX establishment were performed following the EU Directive 2010/63/EU and approved by the 2nd Local Ethics Committee for Animal Experimentation in Warsaw (decision 59/2013).

## CRediT authorship contribution statement

**Marta Zurawska:** Investigation, Methodology, Data curation, Writing – original draft. **Mark Basik:** Conceptualization. **Adriana Aguilar-Mahecha:** Resources. **Michal Dadlez:** Supervision. **Dominik Domanski:** Supervision, Conceptualization, Writing – review & editing.

## Declaration of Competing Interest

The authors declare that they have no known competing financial interests or personal relationships that could have appeared to influence the work reported in this paper.

## Data Availability

Data will be made available on request.

## Appendix Supplementary materials

Image, application 1

Image, application 2

Image, application 3
