# Supplementary material for: A micro-flow, high-pH, reversed-phase peptide fractionation and collection system for targeted and in-depth proteomics of low-abundance proteins in limiting samples
Source: MethodsX. 2023 Jul 31;11:102306. doi: 10.1016/j.mex.2023.102306 (PMC10413349; doi:10.1016/j.mex.2023.102306)
Supplement: Supplementary file 1 [file mmc1.docx]

**Testing of different high-pH buffers in the micro-flow RP LC system.**

For the purpose of micro-flow high-pH RP peptide separation we employed a Waters nanoAcquity UPLC system with a Waters 300 μm x 15 cm, high-pH stable C18 column using an end-capped, silica-based 1.7 µm particle with ‘Ethylene Bridged Hybrid (BEH) technology’, with a usable pH range of 1-12. At a 5 μL/min flow rate, and a column temperature of 40°C, normal pressure was around 6700 ± 300 psi at 2% ACN. We tested nine combinations of mobile phase modifiers, and all but one combination caused eventual clogging of the micro-flow LC system while running only blank injections, usually within two days (Table 1). The compositions most commonly used in standard-flow high-pH separations, employing ammonium formate or ammonium hydroxide with acetonitrile or methanol as the organic solvent, eventually led to system clogging and overpressure. A clogged column was the most common cause, with capillaries from and within the flow controller sometimes also becoming clogged. The less frequently used combinations of triethylamine (TEA) and triethylammonium formate (TEAF) had the same result. This also occurred despite increasing the amount of the aqueous component in the organic solvent (phase B). We finally determined that ammonium bicarbonate, with its lower pH of 8.5, used at 20 mM in phases A and B (with 20% water) resulted in stable system performance for a period of at least eight months.

Potentially such high pressures can momentarily develop locally in the system. It is also known that if the buffer precipitates inside the column it is nearly impossible to dissolve from the pores in the stationary phase, and would explain our observations of only 100% DMSO, but not aqueous solutions, being able to clear a clogged column.

| **Mobile phase A (in H_2_O)** | **Mobile phase B** | **pH** | **Observation** |
| --- | --- | --- | --- |
| 10 mM Ammonium hydroxide | 10 mM Ammonium hydroxide  in ACN | 10 | Column clogged. |
| 10 mM Ammonium hydroxide | 10 mM Ammonium hydroxide  in MeOH | 10 | Column clogged. |
| 10 mM Ammonium hydroxide | 10 mM Ammonium hydroxide  10% H_2_O  in MeOH | 10 | Column clogged. |
| 10 mM Ammonium formate | 10 mM Ammonium formate  in ACN | 10 | Column clogged; ZenFit capillary assembly clogged |
| 10 mM Ammonium formate | 10 mM Ammonium formate  10% H_2_O  in ACN | 10 | Column clogged. |
| 72 mM Triethylamine | 72 mM Triethylamine  20% H_2_O  in ACN | 9.6 | Column clogged. |
| 1% Triethylammonium formate  0.1% Triethylamine | 1% Triethylammonium formate  0.1% Triethylamine  60% H_2_O  in ACN | 10 | Column clogged; capillary from flow-controller clogged |
| 20 mM Ammonium bicarbonate | 20 mM Ammonium bicarbonate  10% H_2_O  in ACN | 9 | Salt precipitate in phase B. |
| **20 mM Ammonium bicarbonate** | **20 mM Ammonium bicarbonate**  **20% H_2_O**  **in ACN** | **8.5** | **No issues after 8 months.** |

Table1. Tested buffers.
