# Supplementary material for: A micro-flow, high-pH, reversed-phase peptide fractionation and collection system for targeted and in-depth proteomics of low-abundance proteins in limiting samples
Source: MethodsX. 2023 Jul 31;11:102306. doi: 10.1016/j.mex.2023.102306 (PMC10413349; doi:10.1016/j.mex.2023.102306)
Supplement: Supplementary file 2 [file mmc2.docx]

**Sample preparation**

1. Reagents and chemicals

Tris (2-carboxyethyl) phosphine hydrochloride (TCEP), chloroacetamide (CAA), HEPES, NaCl, MgCl_2_, CHAPS, and benzonase nuclease were purchased from Sigma-Aldrich (Saint Louis, USA)*.* MS-grade trypsin/Lys-C was purchased from Promega (# V5071, Fitchburg, WI). Peptides with isotopically labeled amino acids on the C-terminus: Arg ^13^C_6_; ^15^N_4_ or Lys ^13^C_6_; ^15^N_2_ (98% isotopic enrichment) were synthesized by JPT Peptide Technologies GmbH as SpikeTides_L (Berlin, Germany).

1. Cell culture

The human breast cancer cell lines: MCF-7 (ATCC^®^ HTB-22^TM^), and MDA-MB-468 (ATCC^®^ HTB-132) were cultured in Dulbecco’s modified Eagle’s (DMEM) high-glucose medium (Sigma Aldrich). The human breast cancer cell lines: EFM-19 (DSMZ ACC 231) was cultured in RPMI-1640 high-glucose medium (Sigma-Aldrich). All cell lines were supplemented with 2 mM l-glutamine (Sigma*-*Aldrich), 10% heat-inactivated fetal bovine serum (Invitrogen), and 1% penicillin/streptomycin (Gibco).

The human breast cancer MCF-7 (ATCC^®^ HTB-22^TM^) cell line was cultured with increasing concentrations of PD0332991 Isethionate (Selleckchem) to reach an IC50 value of 1000 nM. PD0332991 isethionate was solubilized in sterile water at a concentration of 100 mg/mL (174.32 mM). Cell lines were seeded in T175 flasks and incubated at 37 °C and 5% CO_2_ in a sterile biosafety hood, and the medium was changed every 48 h. Cells were grown to 70-80% confluency. Freeze, thaw, passage, and counting protocols were performed according to the American Type Culture Collection (ATCC) methods. Cells were harvested in stocks with 2 × 10^6^ cells suspended in 1 mL of complete medium and 5% DMSO, frozen, and stored at −80 °C until further processing. Cells were harvested at 500 × g for 3 min. and washed twice with cold phosphate buffer solution PBS (Gibco).

1. Cell lysis

For sub-cellular fractionation, cell lysis and extraction of cytoplasmic and nuclear protein fractions was performed using the NE-PER Nuclear and Cytoplasmic Extraction Kit (Thermo Scientific) according to the manufacturer’s instructions. A whole cell lysate was prepared using cold lysis buffer containing 1% sodium deoxycholate in 25 mM HEPES (pH 7.5), incubated at 95^°^C for 5 min. with shaking at 600 rpm; then cooled to RT and incubated with benzonase nuclease at 2.5 U/ μL for 30 min at 4^°^C. Cell debris were removed by centrifugation at 12,000 × g for 10 min. at 4 °C. Different ovarian and colorectal cancer patient-derived xenografts (PDXs) were obtained. PDXs were washed with cold PBS and centrifuged at 300 × g for 3 min. at 4 ^o^C. From each PDX, 50 mg of sample was lysed using a plastic pestle in a 1.5 mL tube using a microtube homogenizer system (VWR 66001-104) in 100 μL of lysis buffer composed of 8 M urea, 50 mM Trizma-base/hydrochloride buffer (Tris-buffer) pH 8.0, 75 mM NaCl, 1 mM MgCl_2_, 1% CHAPS, and benzonase nuclease at 2.5 U/ μL. Lysates were then incubated for 5 min. at 24 ^°^C with shaking at 1000 rpm in a ThermoMixer C Eppendorf shaker, and centrifuged at 14,000 × g for 15 min. at RT. From each type of sample, the extracted proteins were quantified at two dilutions in duplicate using a BCA assay (Thermo Scientific) as per manufacturer’s protocol using the microplate format.

1. Digestion of nuclear and PDX protein samples

Proteins were digested using a modified version of the Single-pot, solid-phase-enhanced sample preparation for proteomics experiments (SP3) protocol [1]. For each sample, 50 μg of proteins was digested in duplicate. Samples were filled up to 100 μL with 50 mM HEPES, pH 7.5, denatured and reduced by adding 3 μL of 0.5 M TCEP, and incubating at 60 ^o^C for 30 min. with shaking at 1000 rpm in a ThermoMixerC Eppendorf shaker. Samples were then alkylated with 7 μL of 0.5 M CAA in water for 40 min. at 25 °C. Next, 10 μL of prepared SP3 beads (according to the SP3 protocol [1]) were added and mixed by gentle pipetting, and 100% ethanol was added to reach a 50% final concentration and incubated at 24 ^o^C for 5 min. at 1000 rpm. Samples were placed in a magnetic rack to discard the supernatant and the beads were rinsed with 500 μL of 80% ethanol. The rinse was repeated three times, and 40 uL of 100 mM ammonium bicarbonate was added to the sample. Proteins were digested with 2 μg of Trypsin/Lys-C reconstituted in 10 μL of 100 mM ammonium bicarbonate (enzyme:protein ratio of 1:25), sonicated in a Bandelin Sonorex water bath sonicator for 30 sec. and incubated at 37 °C for 16 h at 1000 rpm. After the 16 h incubation, samples were sonicated for 30 sec., centrifuged at 4 °C x 1 min. at 16,000 rpm and the supernatant containing the digested peptides was added to a new tube. The mixture of SIS peptides was added to give an amount of individual SIS ranging from 34 to 550 fmol/μg of digest. Samples were dried using a SpeedVac (Labconco) concentrator, and re-suspended in 25 μL of 0.1% formic acid (FA), 2% acetonitrile, sonicated for 5 min. and centrifuged at 14,000 × g for 3 min.

**LC-MS methods**

1. MRM assay development, optimization, and nano-LC-MRM-MS analysis

The selection of tryptic peptides for each target protein for MRM/PRM analysis and optimization of MRM settings using stable isotope-labeled internal standard (SIS) peptides was performed as described previously [2], [3]. Briefly, peptides were selected manually and with the aid of PeptidePicker software following these rules: sequence uniqueness in the human proteome; length between 7 and 25 amino acids; absence of methionine (M) and cysteine (C) residues as both are highly susceptible to oxidation, if possible; high digestion efficiency; absence of known post-translational modifications (PTMs) or significant single nucleotide polymorphisms (SNPs) [4]. Nano-LC-MRM-MS analysis (second dimension; low-pH LC-MRM-MS) was conducted on a Waters Xevo triple-quadrupole (TQ) MS (Waters, Milford, MA) coupled to a Waters nanoAcquity UPLC via a Zspray Nanoflow source with a 10 μm SilicaTip PicoTip emitter (New Objective, MA, USA), a Waters nanoAcquity UPLC BEH130 C18 analytical column (75 μm × 150 mm, 1.7 μm particle size) at 40 °C, and a Waters Symmetry 100 C18 trap-column (180 μm × 20 mm, 5 μm particle size). All the solvents and modifiers were of LC-MS grade. Mobile phase A was 0.1% formic acid (Sigma-Aldrich) in water. Peptides were loaded onto the trap column at a rate of 15 μL/min. and separated at a flow rate of 350 nL/min. using a 60-min LC run, with a gradient of acetonitrile with 0.1% formic acid (mobile phase B) changing from 1 to 10% from 0 to 10 min. and from 10 to 45% from 10 to 40 min, followed by 90% B washing and equilibration. MS parameters included a capillary voltage of 3.5 kV, purge gas flow of 100 L/h, cone gas flow of 5 L/h, NanoFlow gas set at 0.5 Bar, and a source temperature of 150 °C. The initial MRM method targeted 27 proteins, measuring 144 endogenous peptides and 144 complementary SIS peptides using optimized LC-MRM parameters. The endogenous and SIS peptides were each monitored using five fragment ions (1440 scheduled transitions in total) at 0.75-unit resolution, using 10 ms dwell times. The final method was reduced to target 136 peptides and SIS in three 60-min. LC-MRM analyses per non-fractionated sample, while fractions eluted from the high-pH micro-LC separation and concatenated were analyzed using 24 different LC-MRM analyses for 111 endogenous and 111 SIS peptides, each monitored with five fragment ions (1110 transitions in total).

1. Parallel Reaction Monitoring (PRM; SureQuant^TM^) assay development and analysis

The SureQuant^TM^ quantitative scan is an internal standard-triggered (IS)-PRM targeted MS method, performed on a high-resolution (HR) Orbitrap MS instrument, that allows for the quantification of endogenous peptides after a fast MS scan for the detection of SIS peptides. The 27-protein assay was developed as described previously [5]. Briefly, SIS peptides, in a representative matrix, were first characterized by data-dependent acquisition (DDA) with an inclusion list of precursor ions for +2, +3, and +4 charge states to select optimal precursor and product ions. Next, a survey analysis was performed with an inclusion list of optimal charge states for each SIS peptide to define the precursor ion intensity thresholds for MS/MS scan triggering in the final IS-PRM analysis. Dried peptides were reconstituted and analyzed as previously described [6]. using an Evosep LC-One (Evosep Biosystems, Odense, Denmark) coupled to an Orbitrap Exploris 480 MS (Thermo Fisher Scientific, Bremen, Germany). The IS-PRM analysis was performed as described previously [7]. Briefly, data were acquired in positive mode using the following parameters: mass spectra were collected with a scan range of 300–1,500 m/z, an AGC target of 300% (3e^6^), a maximum IT of 50 ms, and a resolution of 120,000. With a 5 second cycle time per MS1 scan, heavy peptides, matching the m/z (within 10 ppm) and the defined intensity threshold on the inclusion list, were isolated (isolation width of 1.0 m/z) and fragmented (nCE:28%) by HCD with a scan range of 100–1,700 m/z, a maximum IT of 10 ms, an AGC target of 1,000% (1e^6^), and a resolution of 7,500. A product ion trigger filter then performs pseudo-spectral matching, triggering only an MS/MS event for the endogenous target peptide at the defined mass offset if n ≥ 5 product ions were detected from the defined list. If triggered, the subsequent light peptide MS/MS scan had the same CE, scan range, and AGC target as the heavy trigger peptide, with a higher maximum injection time and resolution (e.g., max IT:180 ms, resolution: 60,000). The spray voltage was set to 2.1 kV, with a funnel RF level of 40, and a heated capillary temperature of 275 °C.

**Data analysis**

Raw MRM and PRM MS data were analyzed using the Skyline-Daily Ver. 21 software (University of Washington, MacCoss Lab, Department of Genome Sciences, University of Washington). Signals for each peptide were manually inspected to ensure correct peak detection, accurate integration, and interference-free transitions. Equivalent SIS peptide amounts added to all samples enabled the normalization of intensity data between samples in terms of MS signal fluctuations and post-digestion sample processing differences. The amount of each peptide was reported as a peak area ratio which is a ratio of the sum of the areas of transitions for the endogenous peptide compared to the corresponding SIS peptide.

The identification of protein groups and unique peptides in each fraction and in the sample not subjected to fractionation was performed using Proteome Discoverer (PD) version 2.4.0.305. Spectra were searched by Sequest HT against the Homo sapiens SwissProt database (version 2022-06-07, 20.398 sequences). The processing workflow included RC spectrum files, Spectrum Selector, Sequest HT, and Percolator. During the main search, carbamidomethyl (C) was set as a fixed modification, whereas oxidation (M), phosphorylation (STY), acetyl (N-term), and met-loss (M) were set as dynamic modifications. MS and MS/MS mass tolerances were set to 10 ppm and 0.02 Da, respectively. Trypsin digestion was set to full, with a maximum of two missed cleavages allowed. The target–decoy strategy was used to assess FDR, while the target FDR at PSM, peptide and protein levels were set at 0.01.

**References**

[1] Mikulášek, K. *et al.* SP3 Protocol for Proteomic Plant Sample Preparation Prior LC-MS/MS. *Front Plant Sci* **12**, (2021).

[2] Domanski, D. et al. A Multiplexed Cytokeratin Analysis Using Targeted Mass Spectrometry Reveals Specific Profiles in Cancer-Related Pleural Effusions. Neoplasia 18, 399–412 (2016).

[3] Robak, A. et al. Diagnosing pleural effusions using mass spectrometry-based multiplexed targeted proteomics quantitating mid- to high-abundance markers of cancer, infection/inflammation and tuberculosis. Sci Rep 12, 3054 (2022).

[4] Mohammed, Y. *et al.* PeptidePicker: A scientific workflow with web interface for selecting appropriate peptides for targeted proteomics experiments. *J Proteomics* **106**, 151–161 (2014).

[5] Stopfer, L. E. *et al.* High-Density, Targeted Monitoring of Tyrosine Phosphorylation Reveals Activated Signaling Networks in Human Tumors. *Cancer Res* **81**, 2495–2509 (2021).

[6] Jancewicz, I. *et al.* PD-L1 Overexpression, SWI/SNF Complex Deregulation, and Profound Transcriptomic Changes Characterize Cancer-Dependent Exhaustion of Persistently Activated CD4+ T Cells. *Cancers (Basel)* **13**, 4148 (2021).

[7] Kennedy, J. J. *et al.* Internal Standard Triggered-Parallel Reaction Monitoring Mass Spectrometry Enables Multiplexed Quantification of Candidate Biomarkers in Plasma. *Anal Chem* **94**, 9540–9547 (2022).
